# Cold Storage of Rat Hepatocyte Suspensions for One Week in a Customized Cold Storage Solution – Preservation of Cell Attachment and Metabolism

**DOI:** 10.1371/journal.pone.0040444

**Published:** 2012-07-09

**Authors:** Gesine Pless-Petig, Bernhard B. Singer, Ursula Rauen

**Affiliations:** 1 Institut für Physiologische Chemie, Universitätsklinikum Essen, Essen, Germany; 2 Institut für Anatomie, Universitätsklinikum Essen, Essen, Germany; University of Otago, New Zealand

## Abstract

**Background & Aims:**

Primary hepatocytes are of great importance for basic research as well as cell transplantation. However, their stability, especially in suspension, is very low. This feature severely compromises storage and shipment. Based on previous studies with adherent cells, we here assessed cold storage injury in rat hepatocyte suspensions and aimed to find a cold storage solution that preserves viability, attachment ability and functionality of these cells.

**Methods:**

Rat hepatocyte suspensions were stored in cell culture medium, organ preservation solutions and modified TiProtec solutions at 4°C for one week. Viability and cell volume were determined by flow cytometry. Thereafter, cells were seeded and density and metabolic capacity (reductive metabolism, forskolin-induced glucose release, urea production) of adherent cells were assessed.

**Results:**

Cold storage injury in hepatocyte suspensions became evident as cell death occurring during cold storage or rewarming or as loss of attachment ability. Cell death during cold storage was not dependent on cell swelling and was almost completely inhibited in the presence of glycine and L-alanine. Cell attachment could be greatly improved by use of chloride-poor solutions and addition of iron chelators. Using a chloride-poor, potassium-rich storage solution containing glycine, alanine and iron chelators, cultures with 75% of the density of control cultures and with practically normal cell metabolism could be obtained after one week of cold storage.

**Conclusion:**

In the solution presented here, cold storage injury of hepatocyte suspensions, differing from that of adherent hepatocytes, was effectively inhibited. The components which acted on the different injurious processes were identified.

## Introduction

Primary hepatocytes are used for a wide field of basic, pharmacological and toxicological research as well as for cell transplantation and bioartificial liver support systems. Immediately after cell isolation, cells are kept in suspension in buffered salt solutions, organ preservation solutions or cell culture medium at 2–8°C until further use. Usually, this storage lasts between minutes (cell culture) and several hours (cell transplantation); in the case of transfer of cells between labs, shipping times of one day or more may occur. Severe cell injury has been described after cold storage of hepatocyte suspensions in salt solutions, cell culture medium, infusion media or University of Wisconsin (UW) solution [1], [2], [3], thus an improved cold storage protocol for cell suspensions would facilitate delayed use or shipment and enhance cell quality.

Although low temperature is used during storage to protect the cells, it has been shown in adherent cells that cold itself already inflicts cell damage [4]–[7]. This cold-induced cell injury is caused by an increase in intracellular chelatable iron ions [8] and subsequent formation of reactive oxygen species [4], [6], [8]. In adherent rat (but not human) hepatocytes, an additional iron-*in*dependent but chloride-dependent pathway exists, which leads to cell injury mainly during rewarming [9], [10]. The classical view of cold-induced cell injury additionally implicates a disturbed sodium homeostasis and cell swelling [11], [12]; however, in adherent rat hepatocytes we did not find evidence for this [13]. Besides cell death, loss of the ability to attach to cell culture surfaces after cold storage in suspension occurs [1], [3], [14] – rendering part of the stored cells useless for further experiments or decreasing engraftment.

For adherent cells including hepatocytes and for vascular grafts, we recently improved cell survival by modified, mechanism-adapted cold storage solutions [10,15 and B. Akyildiz, S. Straube, U. Rauen, unpublished results]. These solutions contain, most importantly, iron chelators to inhibit iron-dependent formation of reactive oxygen species. Additional components include the amino acids glycine and L-alanine to inhibit hypoxic injury [16], [17], [18], *N*-acetylhistidine as buffer [19], high potassium concentrations, adenosine [10] and, depending on the cell type and application, different chloride concentrations.

In this study, we assessed in the model of primary rat hepatocytes, whether these cold storage solutions or modifications thereof are able to improve cold storage of hepatocytes in suspension and identified the components which improved viability, attachment ability and metabolic function of these cells.

## Materials and Methods

### Chemicals

LK 614, basic solution 1, histidine-tryptophan-ketoglutarate solution (HTK) and *N*-acetylhistidine were kindly provided by Dr. F. Köhler Chemie (Bensheim, Germany). Cold storage solutions (composition see Table 1) are based on the vascular storage solution TiProtec® [15]. Some of these solutions were already tested for cold storage (4°C) of adherent cells [10]. The term ‘iron chelators’ refers to 0.5 mM deferoxamine +20 µM LK 614, the term ‘basic solution’ to the solutions without iron chelators and adenosine.

**Table 1:** Composition of cold storage solutions.

	KH	L-15	Sol. 1	Sol. 2	Sol. 3	Sol. 4	Sol. 5	Sol. 6	Sol. 7
Cl^−^	128.3	145.7	103.1	8.1	103.1	103.1	8.1	8.1	103.1
Lactobionate	–	–	–	95	–	–	95	95	–
Na^+^	143.6	144.6	16	60	104	60	104	16	104
K^+^	5.9	6.2	93	49	5	49	5	93	5
H_2_PO_4_^−^	1.2	1.8	1	1	1	1	1	1	1
SO_4_^2−^	1.2	0.8	–	–	–	–	–	–	–
Mg^2+^	1.2	1.3	8	8	8	8	8	8	8
Ca^2+^	2.5	1.3	0.05	0.05	0.05	0.05	0.05	0.05	0.05
Glycine	–	2.7	10	10	10	10	10	10	–
Alanine	–	2.5	5	5	5	5	5	5	–
α-Ketoglutarate	–	–	2	2	2	2	2	2	2
Aspartate	–	–	5	5	5	5	5	5	5
*N*-Acetylhistidine	–	–	30	30	30	30	30	30	30
Tryptophan	–	0.1	2	2	2	2	2	2	2
Sucrose	–	–	20	20	20	20	20	20	–
Glucose	–	8.3	10	10	10	10	10	10	10
HCO_3_^−^	25	14	–	–	–	–	–	–	–
HEPES	20	–	–	–	–	–	–	–	–
pH	7.35	7.3	7.0	7.0	7.0	7.0	7.0	7.0	7.0
Deferoxamine	–	–	0.5	0.5	0.5	0.5	0.5	0.5	0.5
LK 614	–	–	0.02	0.02	0.02	0.02	0.02	0.02	0.02
Adenosine	–	–	5	5	5	5	5	5	5
Calculated Osmolarity	347	320	311	311	311	311	311	311	275*

KH: Krebs-Henseleit buffer; L-15: Leibovitz L-15 cell culture medium.

All concentrations are given in mM. Cold storage solutions 1–7 without iron chelators (LK 614 and deferoxamine) and without adenosine are referred to as **‘basic solutions’** in the text. L-15 cell culture medium contains amino acids, trace elements etc. not listed in this table (to maintain lucidity) and was additionally supplemented (see Materials and Methods) prior to use. Calculated osmolarity is given in mosm/L.

* Before use, calculated osmolarity was raised to 311 mosm/L (basic solution: 305.2 msom/L) by addition of NaCl.

### Animals

Male Wistar rats (250–350 g) were obtained from the central animal facility of the Universitätsklinikum Essen. Animals received humane care in compliance with the German Law for the Protection of Animals and institutional guidelines, and permission for liver cell isolation was obtained from the local authorities (Landesamt für Natur, Umwelt und Verbraucherschutz Nordrhein-Westfalen, AZ 8.87-50.10.44.08.274).

### Hepatocyte Isolation and Culture

Hepatocytes were isolated as described earlier [6], [20] by *in situ* digestion with 50 U/L collagenase NB 4G (Serva Electrophoresis, Heidelberg, Germany). The liver was then excised, submerged in Krebs-Henseleit buffer (KH; 115 mM NaCl, 25 mM NaHCO_3_, 5.9 mM KCl, 1.2 mM MgCl_2_, 1.2 mM NaH_2_PO_4_, 1.2 mM Na_2_SO_4_, 2.5 mM CaCl_2_, 20 mM HEPES, pH 7.35), the liver capsule gently removed and the cells released by shaking. After sedimentation, a density gradient centrifugation (Percoll in physiological saline, final density 1.09 g/mL, 10 min at 720×g) was performed [20] and the cell pellet was resuspended in KH. Viable cell count was determined via trypan blue exclusion (mean viability 79±6%). For the non-stored control, 1×10^6^ viable cells per well were seeded onto collagen-coated six-well-plates (Sarstedt, Nümbrecht, Germany) in Leibovitz L-15 medium supplemented with 5% (v/v) fetal calf serum, 14.3 mM NaHCO_3_, 8.3 mM D-glucose, 2 mM L-glutamine, 0.1% (w/v) bovine serum albumin, 1 µM dexamethasone, 50 U/mL penicillin and 50 µg/mL streptomycin and cultured at 37°C and 5% CO_2_. After two hours, cell cultures were washed three times with Hanks’ Balanced Salt Solution (HBSS), supplied with fresh culture medium and cultured for another 22 h.

### Cold Storage

For cold storage, 1×10^6^ viable (trypan blue-excluding) cells/mL were resuspended in the respective pre-cooled (4°C) cold storage solution in 1.8 mL cryovials and stored horizontally at 4°C.

### Rewarming/culture after Cold Storage

1 mL cell suspension in the respective cold storage solution was added without further processing to one well of a collagen-coated six-well plate with 2 mL culture medium. After two hours, cell cultures, similar to control cultures, were washed with Hanks’ balanced salt solution to remove unattached cells.

### Determination of Cell Attachment and Morphology

24 h after seeding, cells were washed, cell morphology was assessed and cells were lysed with Triton X-100 (1%). Lactate dehydrogenase (LDH) activity in the lysate of cold-stored cells is expressed as percentage of the respective non-stored control and represents the percentage of adherent, intact cells.

### Cell Viability

#### Cell suspensions

Directly after cell isolation, after cold storage or after 1 h of rewarming, aliquots of the suspension were stained with propidium iodide (PI; 5 µg/mL) for 2 min and analysed using a FACScalibur Flow Cytometer (Becton Dickinson, Franklin Lakes, NJ, USA) at FL3 (λ_exc._ = 488 nm, λ_em._≥670 nm).

#### Adherent cultures

Adherent rat hepatocytes (control and post-storage) were stained with PI (5 µg/mL) after 24 h of culture and assessed by fluorescence microscopy (λ_exc._ = 546±6 nm, λ_em._≥590 nm).

### Determination of Thiobarbituric Acid-reactive Substances

Thiobarbituric acid-reactive substances (TBARS), a marker for lipid peroxidation, were assessed in the incubation solution directly after cold storage as described previously [6]. Concentrations of malondialdehyde equivalents were calculated from a standard curve using 1,1,3,3-tetramethoxypropane.

### Determination of Metabolic Parameters

#### Resazurin conversion

24 h after seeding, conversion of resazurin to resorufin, a general indicator for reductive cell metabolism, was determined as described previously [10]; conversion rate was calculated as percentage of non-stored control cells.

##### Gluconeogenesis

24 h after seeding, cell cultures were incubated in KH buffer with 10 µM forskolin and 20 mM L-lactate at 37°C and 5% CO_2_ for 3 h. The concentration of D-glucose was determined in the supernatant as described previously [10].

#### Urea production

24 h after seeding, cell cultures were incubated in KH buffer with 10 mM D-glucose and 10 mM L-alanine at 37°C and 5% CO_2_ for 3 h. Urea concentration in the supernatant was determined using an enzymatic method based on urease and glutamate dehydrogenase (GLDH). Briefly, 80 µL of the sample were added to 120 µL of reagent yielding final concentrations of 15 mM α-ketoglutarate, 2.5 mM ADP, 0.2 mM NADH and 2.5 U of GLDH in 180 mM TRIS/HCl buffer (pH 8.0). Decrease of NADH absorbance at 340 nm was followed in a plate reader for each sample without (NADH consumption due to ammonia content) and with addition of 1 U urease. Urea concentrations were delineated by the difference between both plots and calculated using an NADH standard curve.

### Statistics

Experiments were performed 6–8 times (see numbers in the respective figure/table legends). If not indicated differently, data are expressed as median and 25/75 percentiles (whiskers in box plots represent 1.5 times the interquartile range, outliers are marked as dots). To limit the influence of inter-experiment variability, pairs/sets compared were always from the same cell isolation. The Friedman test with post-hoc Dunn’s multiple comparison test was used to assess differences between groups. A p-value ≤0.05 was considered as statistically significant.

## Results

### Cell Viability in Hepatocyte Suspensions after Storage at 4°C

**Figure 1:**
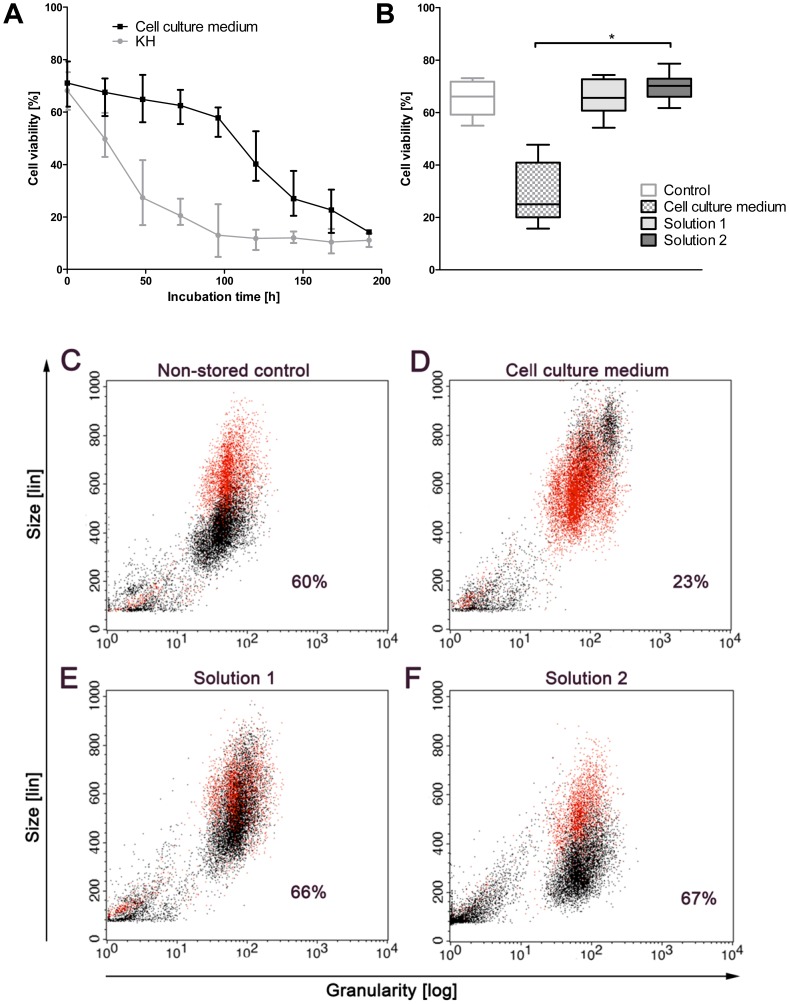
Cell viability after cold storage of primary rat hepatocytes in different solutions. (A) Progression of cell death during cold storage (4°C) in cell culture medium and Krebs-Henseleit-buffer (KH). (B) Cell viability after one week of cold storage in cell culture medium, cold storage solutions 1 and 2 (n = 7;* p = 0.0003). (C-F) Original FACS plots showing cell viability after one week of cold storage. Cells were stained with propidium iodide (PI, 5 µg/mL) and viability was assessed by flow cytometry. C: Non-stored control cells. Cells after cold storage in cell culture medium (D), chloride-rich solution 1 (E) or chloride-poor solution 2 (F). Red dots indicate dead (i.e. propidium iodide-positive) cells. The percentage of viable cells is given in each panel.

Directly after cell isolation, hepatocyte viability as assessed by trypan blue exclusion was 79±6%, and by PI staining 68±5%. When cell suspensions were stored in KH buffer at 4°C, viability decreased fairly rapidly (Fig. 1A). Loss of viability was much slower when cells were stored in cell culture medium. However, after one week of cold storage in cell culture medium, residual viabilities were also below 30% (Fig. 1A, B, D; control cells: 1C). In contrast, in solutions 1 (optimized cold storage solution for adherent human hepatocytes; chloride- and potassium-rich [10]) and 2 (chloride-poor with regard to the chloride-dependent injury of adherent rat hepatocytes [9], with balanced Na^+^/K^+^ concentrations), viabilities after one week of cold storage were still comparable to non-stored control cells (Fig. 1B, E, F).

### Cell Attachment and Cell Morphology after Rewarming

**Figure 2:**
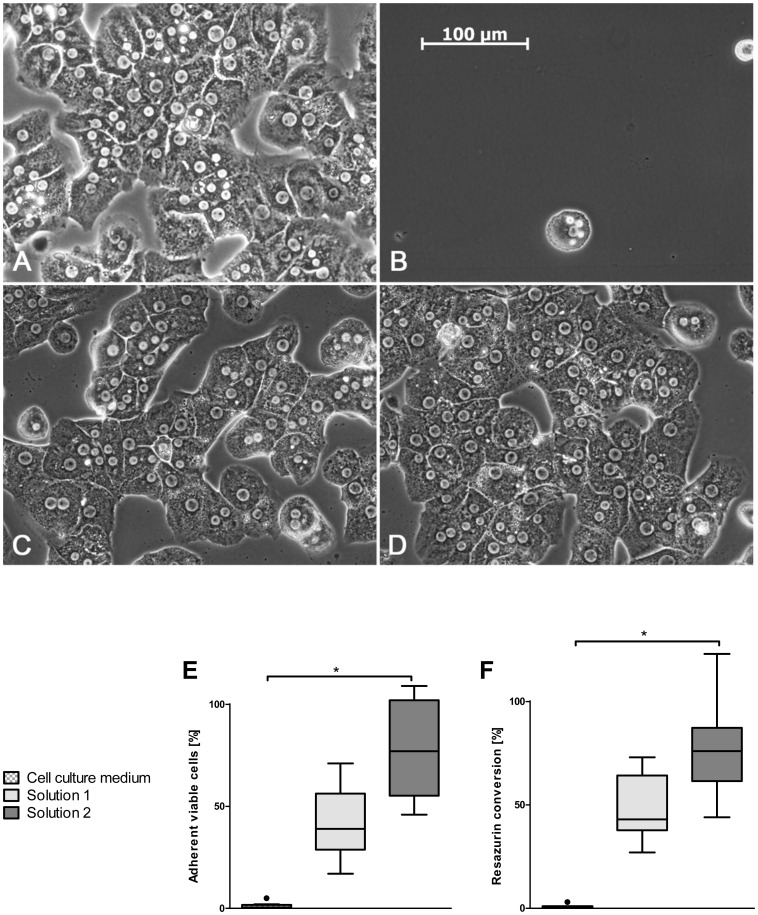
Cell attachment after one week of cold storage in different solutions. (A) Control culture of non-stored primary rat hepatocytes. (B-D) Cell cultures from cell suspensions stored for one week in cell culture medium (B), chloride-rich, potassium-rich solution 1 (C) and chloride-poor solution 2 with balanced Na^+^/K^+^ concentrations (D). (E) Quantification of adherent, viable cells after cold storage and 24 h of cell culture as percentage of cultures of non-stored control cells. (F) Reductive metabolism of cultures obtained from cold-stored cell suspensions, given as percentage of non-stored control cell cultures (n = 8, *p<0.0001).

Control hepatocytes (without cold storage) attached to the cell culture surface to form monolayers and displayed typical trabecular structures after 24 h culture (Fig. 2A). After one week of cold storage of cell suspensions in cell culture medium, cell attachment was virtually absent (Fig. 2B and E). After storage in the chloride-rich solution 1, cell attachment was higher than after storage in cell culture medium (Fig. 2C and E); following storage in chloride-poor solution 2, attachment was even higher (Fig. 2D and E). Cold-stored cells that had attached displayed normal morphology after 24 h of re-culture (Fig. 2C and D) and viability of attached cells was >95% (PI exclusion, data not shown).

### Metabolic Activity after Cold Storage

While control hepatocytes readily reduced resazurin, almost no reductive activity was detectable in cell cultures obtained from cell suspensions after one week of cold storage in cell culture medium (Fig. 2F); reductive metabolism of cultures of cells stored in solution 1 was markedly higher. Cells stored in solution 2 yielded cultures with about 75% of the reductive metabolism of the control cultures, the difference towards cell culture medium being significant.

### Elucidation of the Protective Principles of Solution 2

To explain the superior protection by cold storage solution 2 (Fig. 2D–F), we used modified solutions to assess the influence of the major components separately:

#### Effects of modified solutions on cell viability and cell volume directly after cold storage: Role of ion composition

**Figure 3:**
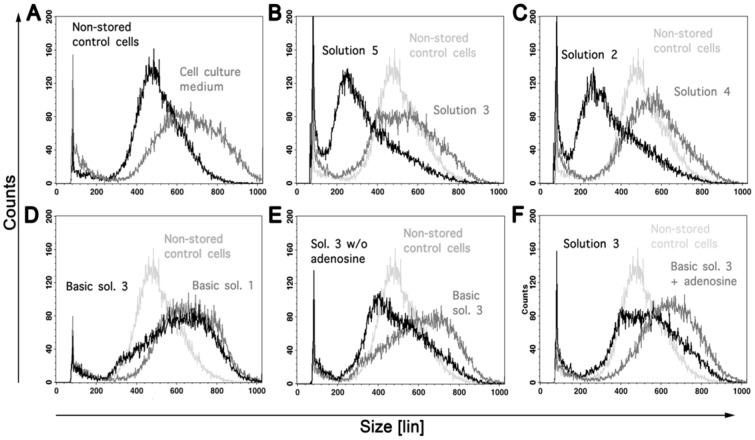
Influence of ion composition and iron chelators on cell volume. Histograms of cell volume distribution as assessed by flow cytometry after one week of cold storage in different solutions. Storage in cell culture medium induced marked cell swelling (A), storage in chloride-rich solutions containing iron chelators slight swelling (B, C). Cell swelling in chloride-rich basic solutions was as pronounced as in cell culture medium, irrespective of sodium concentration (D), substitution of chloride by lactobionate induced marked cell shrinkage (B, C). Cell swelling was inhibited by the addition of iron chelators (E, F; basic solution 3 vs. solution 3 w/o adenosine, solution 3 vs. basic solution 3+ adenosine), but not influenced by addition of adenosine (E, F).

Viability after cold storage was equal in sodium-rich solutions, potassium-rich solutions and solutions with balanced Na^+^/K^+^ concentrations (Table 2, compare solutions 1, 3 and 4 (chloride-rich) or solutions 2, 5 and 6 (chloride-poor)). After cold storage in cell culture medium marked cell swelling (Figs. 1D and 3A) occurred, after storage in chloride-rich solutions 3 and 4 slight cell swelling was observed (Fig. 3B and C). Substitution of chloride by lactobionate (in solutions 2, 5 and 6) did not affect cell viability at the end of cold storage (Table 2, solutions 6 vs. 1, 2 vs. 4 and 5 vs. 3), but induced marked cell shrinkage below the volume of non-stored control cells (Figs. 1F, 3B and C and data not shown).

**Table 2:** Influence of cold storage solution components on cell viability after cold storage.

	Viability after cold storage
Non-stored control	63 (56/72)
Cell culture medium	25 (20/41)
Sol. 1	66 (61/74)
Sol. 2	71 (66/77)*
Sol. 2 without adenosine	70 (66/75)
Sol. 2 (basic)	63 (61/69)
Sol. 3	68 (66/72)
Sol. 3 (basic)	67 (52/68)†
Sol. 4	68 (65/72)
Sol. 5	70 (68/76)*
Sol. 6	72 (69/76)*
Sol. 7 (basic)	18 (16/23)
Sol. 7 (basic) +10 mM glycine +5 mM alanine	42 (28/49)†
Sol. 7 (basic) +20 mM sucrose	21 (20/24)
Sol. 7 (basic) +10 mM glycine +5 mM alanine +20 mM sucrose	56 (45/59)†

‘Basic solution’ refers to solutions without iron chelators (deferoxamine + LK 614) and without adenosine. In supplemented basic solution 7, NaCl addition was reduced to maintain osmolarity. Data is given as median (25/75% percentile).

* significantly different from cell culture medium.

† significantly different from basic solution 7 (n = 6).

#### No role of adenosine

Omitting adenosine from solution 2 had no effect on cell viability and volume directly after cold storage (Table 2 and data not shown).

#### Role of the iron chelators

Although in adherent hepatocytes the strong, hydrophilic iron chelator deferoxamine and the smaller, more membrane-permeable chelator LK 614 applied in the same concentrations as used here provided strong, almost complete inhibition of cold-induced injury [10], the omission of deferoxamine (0.5 mM) and LK 614 (20 µM) did not influence cell viability in rat hepatocyte suspensions directly after cold storage (Table 2, solution 2 without adenosine vs. basic solution 2). However, iron chelators almost completely inhibited cell swelling during cold storage in chloride-rich solutions: cell swelling in basic solutions 1 and 3 was as pronounced as in cell culture medium (Fig. 3D) and the inhibitory effect of the complete solution on cell swelling could be clearly attributed to the iron chelators (Fig. 3E and F).

#### Role of glycine, alanine and sucrose

As cell viability was largely lost after cold storage in cell culture medium but not in modified TiProtec solutions, including the sodium chloride-rich solution 3 and basic solution 3 (Table 2), we omitted glycine, alanine and sucrose from basic solution 3, resulting in basic solution 7. After cold storage in basic solution 7, cell viability was even below the viability of cells stored in cell culture medium (Table 2). Re-addition of glycine and alanine to basic solution 7 significantly increased hepatocyte viability after cold storage (Table 2), re-addition of sucrose alone had only a small effect on viability (Table 2). No effect of glycine, alanine and sucrose could be observed on cell volume after cold storage (data not shown).

#### Effects of modified solutions on cell attachment: Role of the main anion

**Figure 4:**
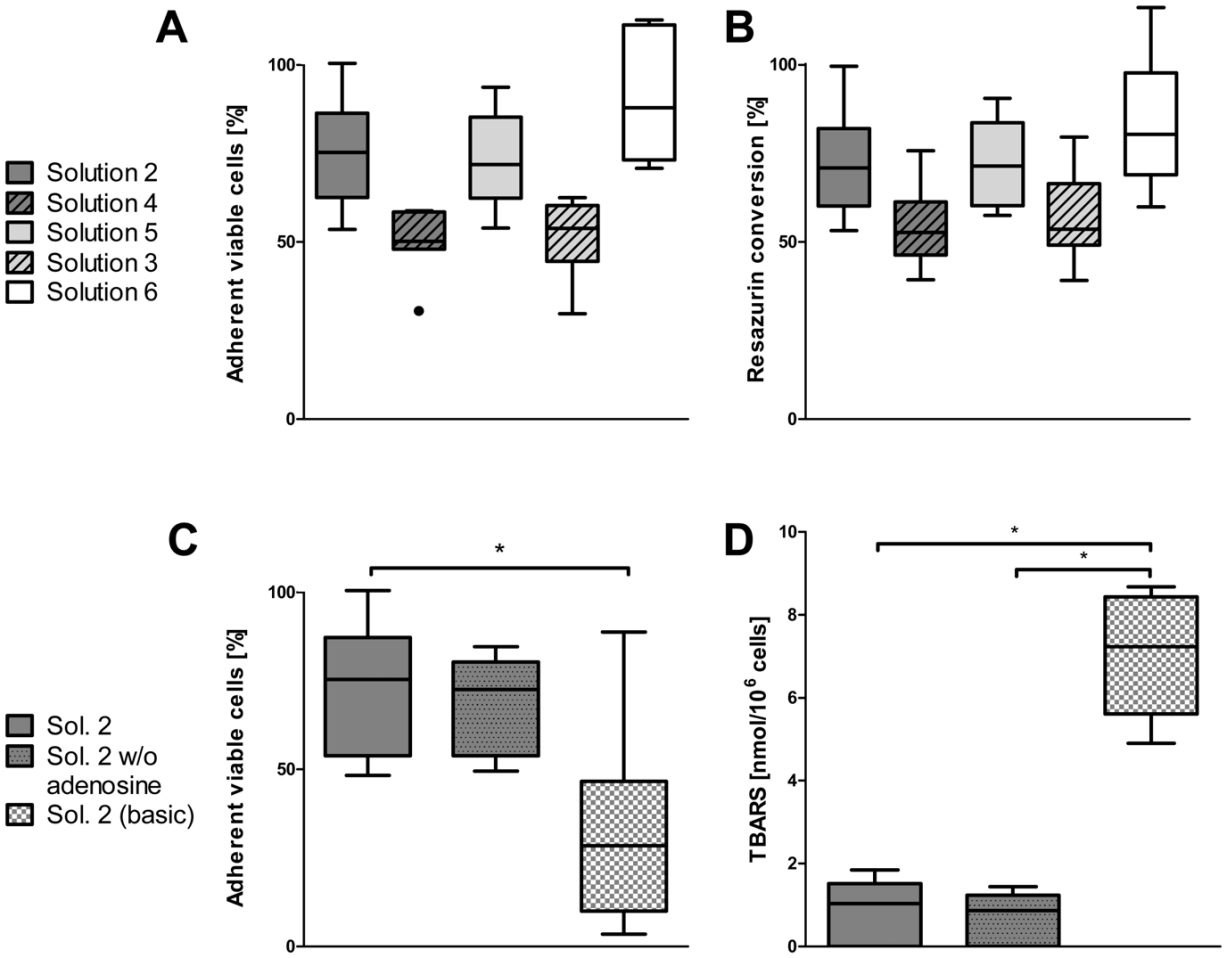
Influence of ion composition and iron chelators on cell attachment and metabolism. Influence of ion composition of cold storage solutions after one week of cold storage on cell adherence (A) and metabolism (B; n = 6). Dark grey bars: solutions with balanced Na+/K+ concentrations, light grey bars: sodium-rich solutions, open bars: potassium-rich solutions; striped bars: chloride-rich solutions. Influence of iron chelators in the cold storage solution on cell attachment (C; p = 0.0303) and lipid peroxidation (D; p = 0.0017; compare dotted bar, solution 2 w/o adenosine and checkered bar, solution 2 (basic); n = 6).

Although chloride did not affect viability after cold storage, the superiority of solution 2 over solution 1 in cell attachment (Fig. 2D–F) suggested that low chloride concentrations during cold storage might improve cell attachment. Systematical testing confirmed this hypothesis: cells stored in the chloride-poor solution 2 yielded a markedly higher attachment rate than cells stored in the respective chloride-rich analogue, solution 4 (Fig. 4A). Cold storage in chloride-poor solution 5 was largely superior to cold storage in the respective chloride-rich solution 3. Reductive metabolism was also higher after cold storage in chloride-poor solutions (solutions 2, 5 and 6, Fig. 4B).

#### Role of the main cation

Storage in potassium-rich solution 6 resulted in slightly higher cell attachment compared to sodium-rich solution 5 and solution 2 (balanced Na^+^/K^+^ concentrations; Fig. 4A), which was also reflected in the maintenance of resazurin conversion ability (Fig. 4B).

#### No role of adenosine

Adenosine had virtually no effect on cell attachment (Fig. 4C).

#### Role of iron chelators

Although iron chelators did not influence cell viability in cell suspensions directly after cold storage (Table 2), their presence resulted in a markedly higher number of attached cells after 24 h of culture (Fig. 4C; solution 2 without adenosine vs. basic solution 2) and markedly higher resazurin conversion (data not shown). Assessment of TBARS confirmed that lipid peroxidation occurred during cold storage of hepatocyte suspensions in basic solution 2 and was almost completely inhibited in the presence of iron chelators during cold storage (Fig. 4D).

### Effects of Solution Components on Cell Viability during Rewarming

The superior cell attachment after cold storage in the presence of iron chelators and/or in the absence of chloride was not caused by the inhibition of rewarming injury: During one hour of rewarming of cell suspensions in cell culture medium/cold storage solution (2∶1; 37°C, 5% CO_2_), the additional loss of viability was relatively small, between 10 and 20% in all solutions, irrespective of the presence of adenosine, iron chelators, chloride or of sodium or potassium concentrations (data not shown).

### Comparison of Solution 2 with Established Organ Preservation Solutions

**Figure 5:**
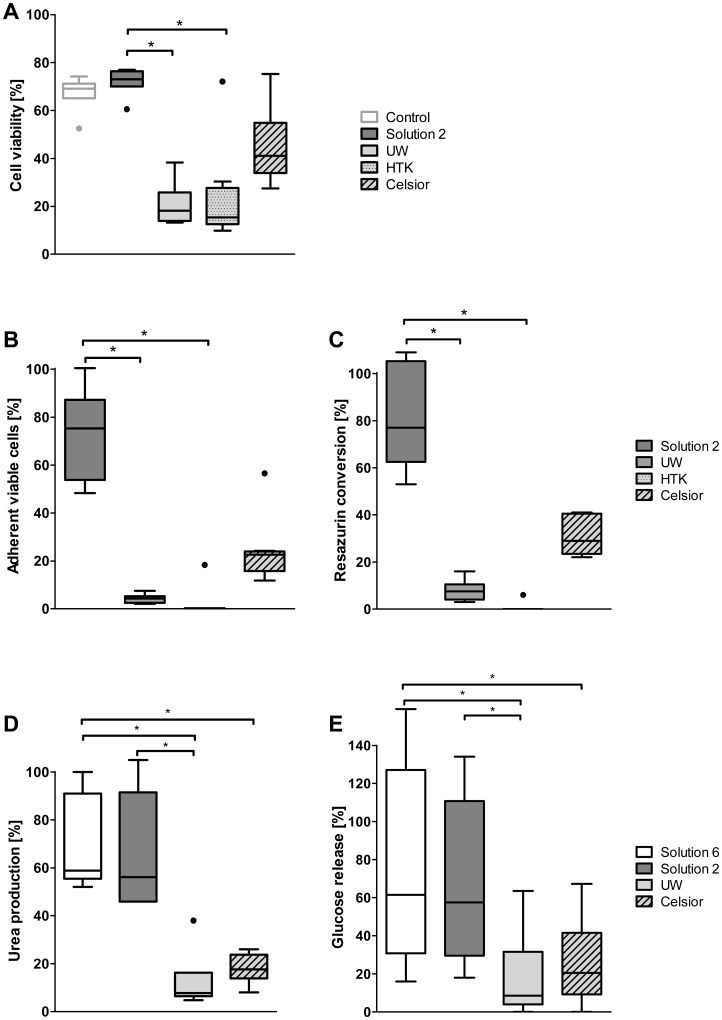
Comparison of solutions 2 and 6 to established organ preservation solutions. Viability (A), cell attachment (B) and resazurin conversion (as measure for general reductive metabolism, C) after one week of cold storage in solution 2, University of Wisconsin solution (UW), histidine-tryptophan-ketoglutarate solution (HTK) and Celsior (n = 8). Urea production (D) and forskolin-triggered glucose release (E) of cultures from cells stored in solutions 2 and 6, UW and Celsior for one week (n = 6). Activities are expressed as percentage of cultures from non-stored control cells; *p≤0.0001.

Finally, the new cold storage solution was compared to other solutions used for cell preservation, namely the organ preservation solutions UW, HTK and Celsior. After cold storage, hepatocyte viability was markedly decreased in all other preservation solutions compared to solution 2 after one week of cold storage (Fig. 5A). Consequently, cell attachment was markedly higher after cold storage in solution 2 than after cold storage in organ preservation solutions (Fig. 5B). These results were confirmed by the data on cell morphology (data not shown) and reductive metabolism (Fig. 5C).

### Maintenance of Hepatocyte-specific Functions after Cold Storage

Cells stored for one week at 4°C in solution 2 or solution 6 retained about 60% of the capacity for urea production of unstored control cells, while in cells stored in UW or Celsior solution, urea production after cold storage was reduced to below 20% of controls (Fig. 5D). Forskolin-triggered glucose release of cultures from cells stored in solutions 2 and 6 was also about 60% of that of unstored control cells, whereas after cold storage in UW and Celsior solution, glucose release was at about 10–20% (Fig. 5E).

## Discussion

Our results show that the injury occurring to rat hepatocytes stored in suspension at 4°C is only partly evident directly after cold storage (Fig. 1B), but becomes articulately evident as lack of cell attachment (Fig. 2A–E). However, cell survival and attachment rate could be greatly improved by using a chloride-poor cold storage solution containing glycine, alanine and iron chelators, in which glycine and alanine inhibited the injury occurring during cold storage itself while the absence of the anion chloride and the presence of iron chelators during cold storage largely increased the attachment rate.

### Cold-induced Cell Injury – Classical View

Classically, cold-induced cell injury has been attributed to cold-induced inhibition of the Na^+^/K^+^-ATPase, which was thought to cause cellular sodium and chloride accumulation, cell swelling and finally cell death [11], [12]. However, in adherent cells we showed that intracellular sodium and chloride contents decreased during normoxic cold storage and that sodium accumulation is a phenomenon associated with hypoxia rather than hypothermia [13]. Furthermore, cold-induced death of adherent cells turned out to be of an apoptotic rather than necrotic type [5], [6], thus likely to be associated with cell shrinkage rather than swelling [21]. With cells in suspension, cell swelling after storage was observed in all iron chelator-free, chloride-containing solutions – the concentration of sodium did not affect cell swelling (Fig. 3D). Substitution of chloride by lactobionate induced marked cell shrinkage (Fig. 3B and C). Still, neither cell swelling nor cell shrinkage by itself was associated with cell death during cold storage. Although the attachment rate was largely enhanced after cold storage in chloride-poor new solutions (Fig. 4A), the protective principle ‘chloride-poor’ alone was not sufficient for good preservation – as the low attachment rate in the chloride-poor UW and Celsior solutions (Fig. 5A–C) and in basic solution 2 (Fig. 4C) showed.

### Role of Chelatable Iron Ions in Cold-induced Cell Injury

Diverse studies have shown that in adherent rat hepatocytes, as in many other adherent cell types, iron-dependent injury is the major component of cold-induced cell injury [4], [5], [6], [8]. It already occurs during cold storage itself, is aggravated during the early rewarming phase [6], [9] and is triggered by a pronounced increase in cytosolic chelatable iron ions [8], [22], [23], leading to formation of highly reactive oxygen species and subsequent oxidative injury mainly targeting the mitochondria [4], [5], [24]. This injury is effectively inhibited by iron chelators. Surprisingly, in rat hepatocyte suspensions hardly any protective effect of iron chelators could be observed during cold storage itself (Table 2), although lipid peroxidation was inhibited (Fig. 4D). However, the presence of iron chelators inhibited cell swelling in chloride-rich solutions (Fig. 3E and F) and largely improved cell attachment (Fig. 4C). Thus, iron-dependent injury appears to be less pronounced in hepatocyte suspensions, not leading to cell death within 24 h (as seen in adherent hepatocytes), but causing lipid peroxidation (Fig. 4D) and thereby likely causing sublethal membrane alterations/increased membrane permeability compromising intracellular ion homeostasis (cell swelling, Fig. 3E, F) and inducing functional damage that obviously prevents cell attachment.

### Protection of Cell Suspensions during Cold Storage Itself

Neither the cation nor the anion composition of the solution nor the iron chelators affected cell death during cold storage itself. The main components identified in this study to account for the protective effect during cold incubation were glycine and alanine. Sucrose provided minor protection. Marsh et al. found a protective effect of glycine in rat hepatocytes stored in suspension [25], which they attributed to its role in glutathione synthesis. However, glycine and alanine are also potent inhibitors of hypoxic cell injury [18] and might target hypoxic rather than hypothermic cell injury in cell suspensions. Alternatively, cold-induced mitochondrial impairment, e.g. due to cold-induced mitochondrial fragmentation [10], [26], might give rise to energy deficiency-induced injury, against which glycine has also been shown to provide protection [17].

### Cell Viability vs. Cell Attachment after Cold Storage

In most studies, cell viability in suspension is assessed directly after cold storage or after a short rewarming phase in suspension [27], [28], [29]; although this parameter is very insensitive, loss of cell viability has been described to occur after 24–120 h. Compared to adherent rat hepatocytes [6], rewarming injury was not very pronounced in cell suspensions in the current study. Furthermore, since the crude cell suspension was used for the experiments, cell death during rewarming is likely to be partly due to cell damage already initiated during the cell isolation process. As this overlaps with the diverse cold-induced processes, it is not surprising that no inhibitory effect of single interventions on the relatively small rewarming component of the total injury could be elucidated here.

Although little lethal cell injury became evident during rewarming/reculturing, cell attachment proved to be a cell function severely compromised after cold storage (compare figures 1B and 2E). This is possibly due to the complex cytoskeletal reorganizations required during this process; cell attachment has not yet been reported after cold storage periods exceeding 72 h [1], [2], [30]. Here, we showed that crude cell suspensions stored in the new hepatocyte storage solutions (solutions 2 and 6) for even one week were still able to form normal adherent cell cultures – without further processing of the cell suspensions and with a cell function close to cultures from non-stored control cells, a functionality not yet described for stored hepatocyte suspensions.

### Conclusion and Perspectives

Normal cell cultures could be obtained from primary rat hepatocyte suspensions after one week of cold storage in the new chloride-poor solution containing alanine, glycine, sucrose and iron chelators. Future studies will prove the transferability of the results to other primary cell types and/or species, especially with regard to human hepatocytes used for cell transplantation.
